# First record of the genus *Anthomalachius* Tshernyshev, 2009 (Coleoptera, Malachiidae) from China

**DOI:** 10.3897/BDJ.12.e141874

**Published:** 2024-12-30

**Authors:** Junbo Tong, Sergei E. Tshernyshev, Yuxia Yang, Haoyu Liu

**Affiliations:** 1 The Key Laboratory of Zoological Systematics and Application, School of Life Science, Institute of Life Science and Green Development, Hebei University, 071002, Baoding, China The Key Laboratory of Zoological Systematics and Application, School of Life Science, Institute of Life Science and Green Development, Hebei University, 071002 Baoding China; 2 Institute of Systematics and Ecology of Animals, Siberian Branch of the Russian Academy of Sciences, Frunze Street 11, 630091, Novosibirsk, Russia Institute of Systematics and Ecology of Animals, Siberian Branch of the Russian Academy of Sciences, Frunze Street 11, 630091 Novosibirsk Russia; 3 Tomsk State University, Lenina prospekt 36, 634050, Tomsk, Russia Tomsk State University, Lenina prospekt 36, 634050 Tomsk Russia

**Keywords:** Cleroidea, Malachiini, melyrid lineage, new faunistic record, taxonomy

## Abstract

**Background:**

*Anthomalachius* Tshernyshev, 2009 is a small genus belonging to the tribe Malachiini in the family Malachiidae of the order Coleoptera, with six currently known species: *A.spinosus* (Erichson, 1840) from Central, Eastern and Southern Europe and North Africa; *A.strangulatus* (Abeille de Perrin, 1885) from Central Europe to Central Asia; *A.davoodi* Ezzatpanah, 2011 from Iran; *A.senylia* (Tshernyshev, 2000) from Uzbekistan and Kyrgyzstan; *A.foveatus* (Medvedev, 1964) from Kazakstan, Russia and Mongolia; *A.pseudospinosus* (Medvedev, 1964) from Kazakhstan and Turkey.

**New information:**

In this study, *Anthomalachius* Tshernyshev, 2009 is newly recorded from China upon the discovery of *A.pseudospinosus* (Medvedev, 1964) from Xinjiang Autonomous Region. The adult morphological characters are re-described in detail with illustrations of external appearance and relevant characters. The female ovipositor, pygidium and ultimate abdominal ventrite of *A.pseudospinosus* are illustrated and described for the first time.

## Introduction

The genus *Anthomalachius* was proposed by Tshernyshev (2009) on the basis of five species separated from the genus *Clanoptilus* Motschulsky, 1854 of the family Malachiidae (Majer 2002, Mayor 2007, Bocakova et al. 2012). *Anthomalachius* can be distinguished from *Clanoptilus* by the black and simple antennae, elongate pronotum with rough surface and thinner aedeagus (Tshernyshev 2009, Franzini and Morin 2019). In contrast, *Clanoptilus* has antennae somewhat yellow with specialised male structures, transverse pronotum with smooth surface and rather robust aedeagus (Tshernyshev 2009, Franzini 2019).

To date, the genus *Anthomalachius* is comprised of six species distributed from Portugal and Morocco in the west to Mongolia in the east (Tshernyshev 2009, Ezzatpanah 2011). In the present study, some specimens of this genus were discovered from China and identified as *A.pseudospinosus* (Medvedev, 1964), which represents the first record of *Anthomalachius* from the Chinese fauna. Hence, a more detailed description of the species, as well as illustrations of external appearance and specialised male characters with distribution map are provided. The female ovipositor, pygidium and ultimate abdominal ventrite of *A.pseudospinosus* are illustrated and described for the first time.

## Materials and methods

In this study, Malachiidae beetles are considered as a family (Majer 1994, Majer 2002, Mayor 2007, Bocakova et al. 2012), instead of a subfamily within Melyridae (e.g. Gimmel et al. (2019)).

Terminology of terminalia morphology is according to Lawrence et al. (2010), namely pygidium for apical tergite, ultimate abdominal ventrite for apical sternite and endophallus for the inner sac of the aedeagus.

For dissection, the specimen had its abdomen detached and soaked in 10% solution of sodium hydroxide (NaOH) by boiling for several minutes. The ovipositor was dyed with haematoxylin. Genitalia were dissected, cleaned and transferred to glycerol on slides, then photographed with a LEICA DFC450 digital camera attached to the LEICA M205 A microscope. LAS V.4.7 software was used to capture genitalia images. External morphology was observed with a Nikon SMZ1500 stereomicroscope. Images of adults were taken with a Canon EOS 80D digital camera and focus-stacked in Helicon Focus 7. The final plates were prepared in Adobe Photoshop CC 2020.

The specimens examined in this study are deposited in Institute of Zoology, Chinese Academy of Sciences, Beijing, China (IZAS) and Museum of Hebei University, Baoding, China (MHBU).

## Taxon treatments

### Anthomalachius

Tshernyshev, 2009

23EE7D7B-C73E-5EF7-9064-C24D72162031

Anthomalachius Tshernyshev, 2009 - Tshernyshev (2009): 24.
Cyrtosus strangulatus Abeille de Perrin, 1885

#### Diagnosis

**Figure 1.**
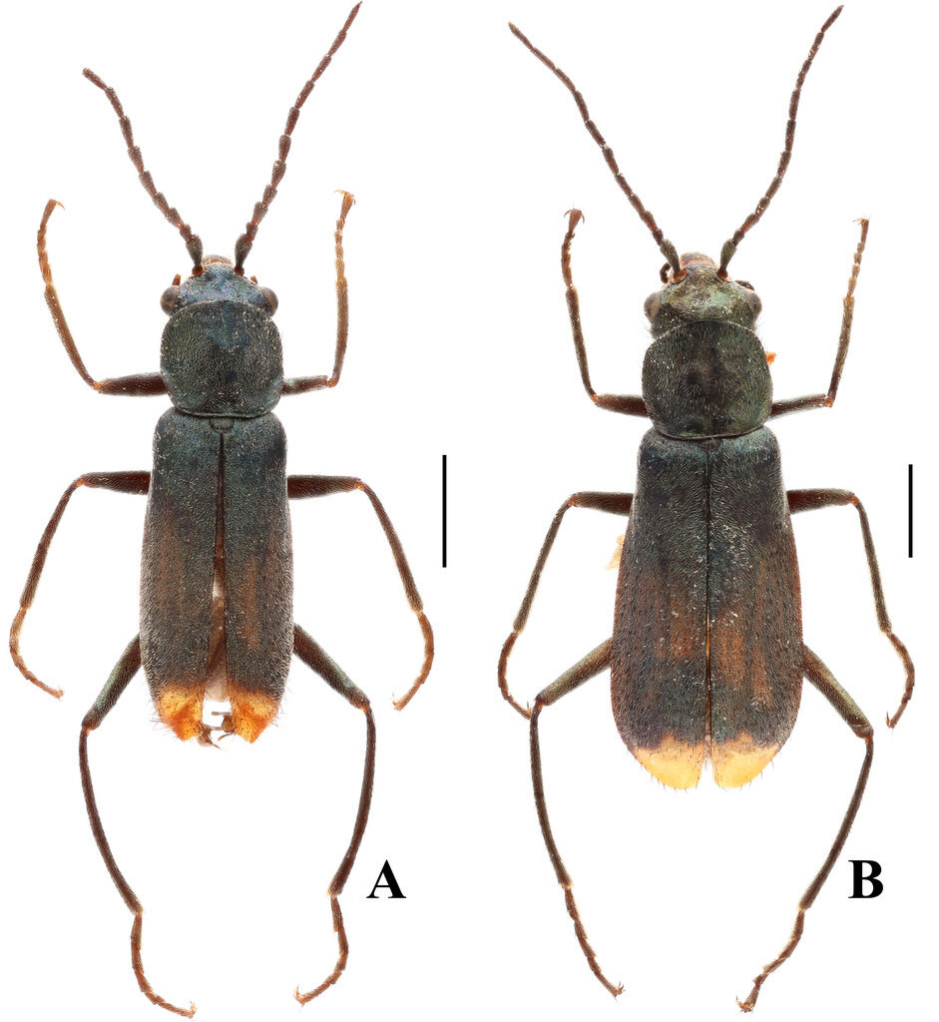
*Anthomalachiuspseudospinosus* (Medvedev, 1964), habitus: **A** male, dorsal view; **B** female, dorsal view. Scale bars: 1.0 mm.

**Figure 2.**
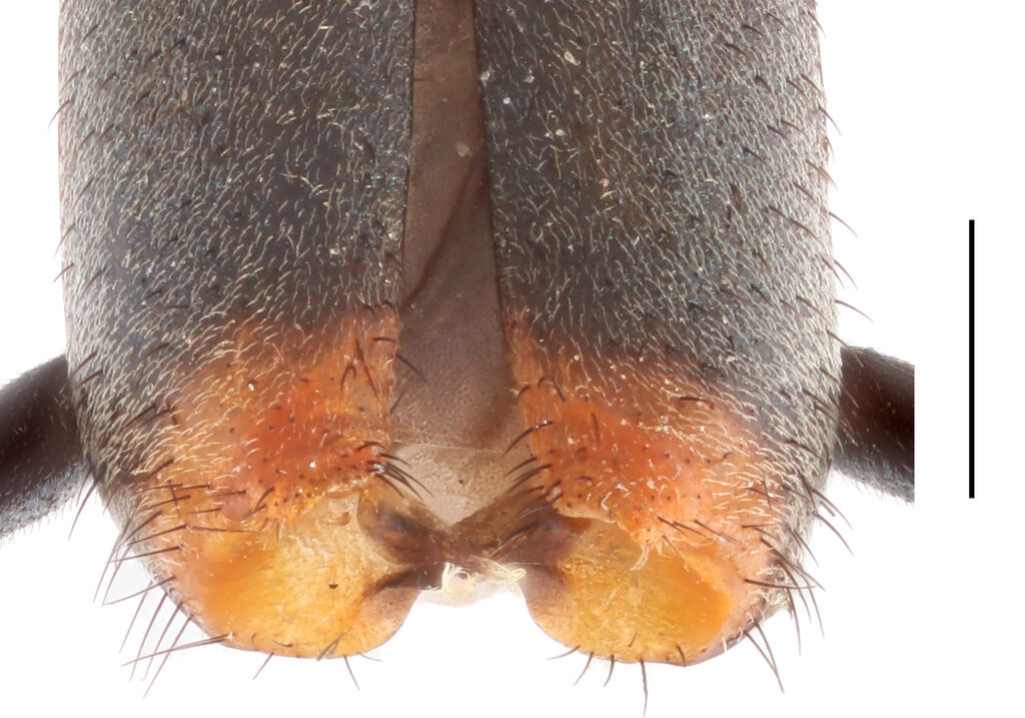
*Anthomalachiuspseudospinosus* (Medvedev, 1964), male, elytral apices. Scale bars: 1.0 mm.

Body medium-sized to large, 4.0–5.5 mm in length. Antennae completely black (Fig. 1A–B). Pronotum elongate with distinct depressions at posterior angles (Fig. 4A–B). Pronotum, scutellar shield and elytra black with metallic lustre, except the elytral apices yellow or orange-yellow. Elytral apices distinctly depressed with lamellate appendages in males (Fig. 2). Aedeagus slender (Fig. 4).

#### Distribution

*Anthomalachius* Tshernyshev, 2009 is a widespread genus in the Palearctic, occurring from Portugal and Morocco in the west to Xinjiang Autonomous Region of China (new faunistic record) and Mongolia in the east (Tshernyshev 2009).

### Anthomalachius pseudospinosus

(Medvedev, 1964)

64F8F82A-5A4F-5CA2-AF0A-05FB7258AECD

Malachius pseudospinosus Medvedev, 1964 - Medvedev (1964): 156; Medvedev (1980): 117 (type locality: Kazakhstan).
Anthomalachius pseudospinosus (Medvedev, 1964): Tshernyshev (2009): 29.

#### Materials

- **Type status:** Other material. **Occurrence:** recordedBy: Shijun Ma; sex: 3 females; lifeStage: adult; occurrenceID: F6F52111-1D86-59E2-9C68-9BA111398C79; **Location:** country: China; stateProvince: Xinjiang; locality: Tacheng; **Event:** year: 1955; month: 7; day: 22; **Record Level:** institutionID: Institute of Zoology, Chinese Academy of Sciences; institutionCode: IZAS
- **Type status:** Other material. **Occurrence:** recordedBy: Shijun Ma; sex: 2 females; lifeStage: adult; occurrenceID: 19C4AD13-1C41-5A74-8975-A2D06ABE1179; **Location:** country: China; stateProvince: Xinjiang; county: Emin; locality: Emin River; **Event:** year: 1955; month: 7; day: 22; **Record Level:** institutionID: Institute of Zoology, Chinese Academy of Sciences; institutionCode: IZAS
- **Type status:** Other material. **Occurrence:** recordedBy: Xiongyi Yang; sex: 1 male; lifeStage: adult; occurrenceID: C5519162-0015-5F11-8500-DFC3488E92A2; **Location:** country: China; stateProvince: Xinjiang; county: Qinghe; **Event:** year: 1956; month: 8; day: 2; **Record Level:** institutionID: Institute of Zoology, Chinese Academy of Sciences; institutionCode: IZAS
- **Type status:** Other material. **Occurrence:** recordedBy: collector unknown; sex: 3 females; lifeStage: adult; occurrenceID: C00F1CDC-2D2B-5CA5-BA20-2AF2620DB5A3; **Location:** country: China; stateProvince: Xinjiang; county: Qinghe; locality: Aerxiate; **Event:** year: 1956; month: 8; day: 2; **Record Level:** institutionID: Institute of Zoology, Chinese Academy of Sciences; institutionCode: IZAS
- **Type status:** Other material. **Occurrence:** recordedBy: Chunpei Hong; sex: 7 males, 5 females; lifeStage: adult; occurrenceID: 8CD71849-D176-5498-A855-6E4CD5B3A324; **Location:** country: China; stateProvince: Xinjiang; county: Wulumuqi; locality: Wulabai; **Event:** year: 1957; month: 6; day: 2; **Record Level:** institutionID: Institute of Zoology, Chinese Academy of Sciences; institutionCode: IZAS
- **Type status:** Other material. **Occurrence:** recordedBy: Guang Wang; sex: 1 female; lifeStage: adult; occurrenceID: 0F597C9B-A0D5-5002-81B0-13AFD57B5AF0; **Location:** country: China; stateProvince: Xinjiang; county: Shihezi; locality: Manasi; verbatimElevation: 510 m; **Event:** year: 1957; month: 6; day: 7; **Record Level:** institutionID: Institute of Zoology, Chinese Academy of Sciences; institutionCode: IZAS
- **Type status:** Other material. **Occurrence:** recordedBy: Guang Wang; sex: 3 females; lifeStage: adult; occurrenceID: 8FE13DA6-C456-5882-A40E-BB4FCFDCB694; **Location:** country: China; stateProvince: Xinjiang; county: Shihezi; locality: Manasi; verbatimElevation: 415-550 m; **Event:** year: 1957; month: 6; day: 8; habitat: Clover field; **Record Level:** institutionID: Institute of Zoology, Chinese Academy of Sciences; institutionCode: IZAS
- **Type status:** Other material. **Occurrence:** recordedBy: Guang Wang; sex: 1 female; lifeStage: adult; occurrenceID: 9213E76F-910F-544B-A745-C28626E50A82; **Location:** country: China; stateProvince: Xinjiang; county: Shihezi; locality: Manasi; verbatimElevation: 460-510 m; **Event:** year: 1957; month: 6; day: 8; habitat: Cotton field; **Record Level:** institutionID: Institute of Zoology, Chinese Academy of Sciences; institutionCode: IZAS
- **Type status:** Other material. **Occurrence:** recordedBy: Guang Wang; sex: 1 female; lifeStage: adult; occurrenceID: 4D36D9C8-CBDF-5C05-8252-056F23B94A2D; **Location:** country: China; stateProvince: Xinjiang; county: Zhaosu; verbatimElevation: 1120-1620 m; **Event:** year: 1957; month: 8; day: 7; **Record Level:** institutionID: Institute of Zoology, Chinese Academy of Sciences; institutionCode: IZAS
- **Type status:** Other material. **Occurrence:** recordedBy: collector unknown; sex: 1 female; lifeStage: adult; occurrenceID: 190DA624-1382-5E0A-849F-836BCE4E86AD; **Location:** country: China; stateProvince: Xinjiang; county: Hutubi; **Event:** year: 1959; month: 7; day: 8; habitat: Mung bean field; **Record Level:** institutionID: Institute of Zoology, Chinese Academy of Sciences; institutionCode: IZAS
- **Type status:** Other material. **Occurrence:** recordedBy: Guodong Ren; sex: 1 female; lifeStage: adult; occurrenceID: 57A0A65C-3EFE-53D7-9602-BB0D567637B3; **Location:** country: China; stateProvince: Xinjiang; county: Beitun; locality: Mu'erchang; **Event:** year: 1992; month: 8; day: 8; **Record Level:** institutionID: Museum of Hebei University; institutionCode: MHBU

#### Description

**Male.** Length of body 4.2–5.0 mm, width at widest part of elytra 1.4–1.8 mm and at the base of elytra 1.0–1.2 mm.

**Figure 3.**
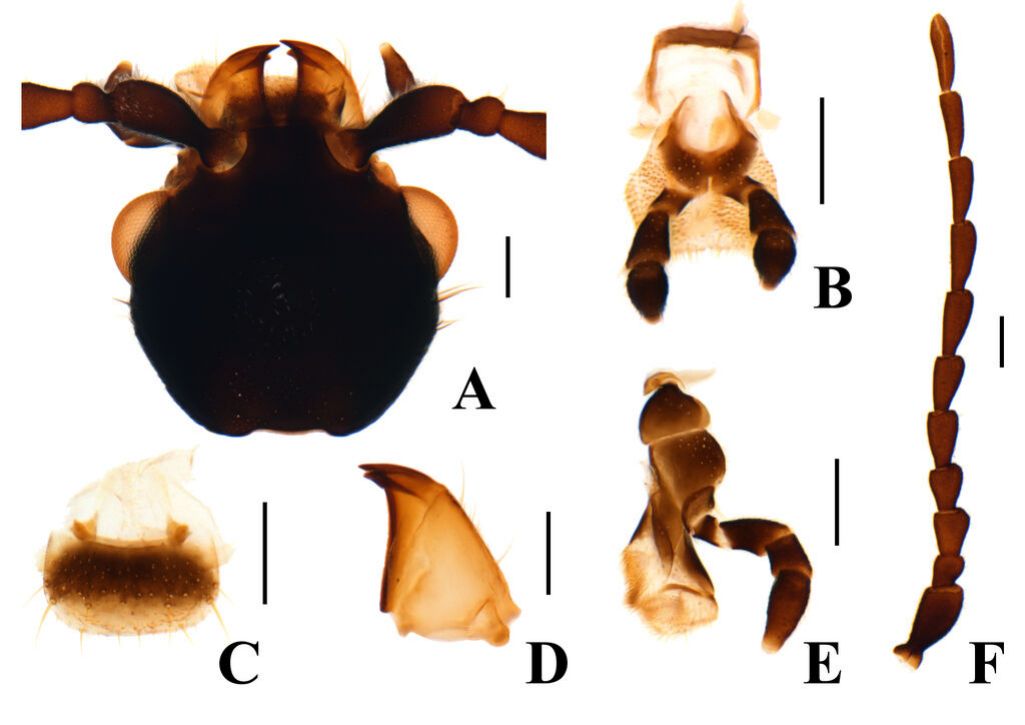
*A.pseudospinosus* (Medvedev, 1964), male. **A** head; **B** labium; **C** labrum; **D** mandible; **E** maxilla; **F** antenna. Scale bars: 0.2 mm.

Body mostly black with metallic lustre, except clypeus, mandibles, genae and front side of labrum yellow and the elytral apices orange or yellow (Fig. 1A and Fig. 3A). Vesicles yellow. Dorsum with double pubescence consisting of adpressed pubescence and sparse black stiff bristles. Sculptures inconspicuous.

Head almost as wide as pronotum. Frons with a shallow depression. Labial palps with 3 palpomeres, apical palpomere suboval (Fig. 3B). Clypeus distinct and membranous (Fig. 3C). Mandible triangular, with a subapical tooth just behind apical tooth (Fig. 3D). Maxilla with cardo short; palpomere Ⅱ elongate; III subquadrate; Ⅳ subconical (Fig. 3E). Antennae long, reaching the middle of elytra; antennomere Ⅰ club-shaped; Ⅱ small and subquadrate; Ⅲ–Ⅹ trapezoidal; Ⅺ elongated oval (Fig. 3F).

Pronotum slightly transverse, anterior margin arcuate, posterior margin straight, all angles rounded, with distinct depressions at posterior angles.

Scutellar shield small, transverse, with smoothed edges.

Elytra subparallel, base of elytra slightly wider than pronotum. Humeri distinct, slightly protruding. Elytral apices distinctly depressed, with lamellate vertical appendages provided with small curved plate, bearing a tuft of long curved hairs distally (Fig. 2).

Hind wings normally developed.

Legs slender. Hind femora reaching elytral apices. All tibiae thin and straight. All tarsi with 5 tarsomeres; tarsomere II of fore tarsi simple, lacking specialised comb; Ⅴ longest and Ⅳ shortest in all legs. Claws thin and sharp, with membrane at base.

**Figure 4.**
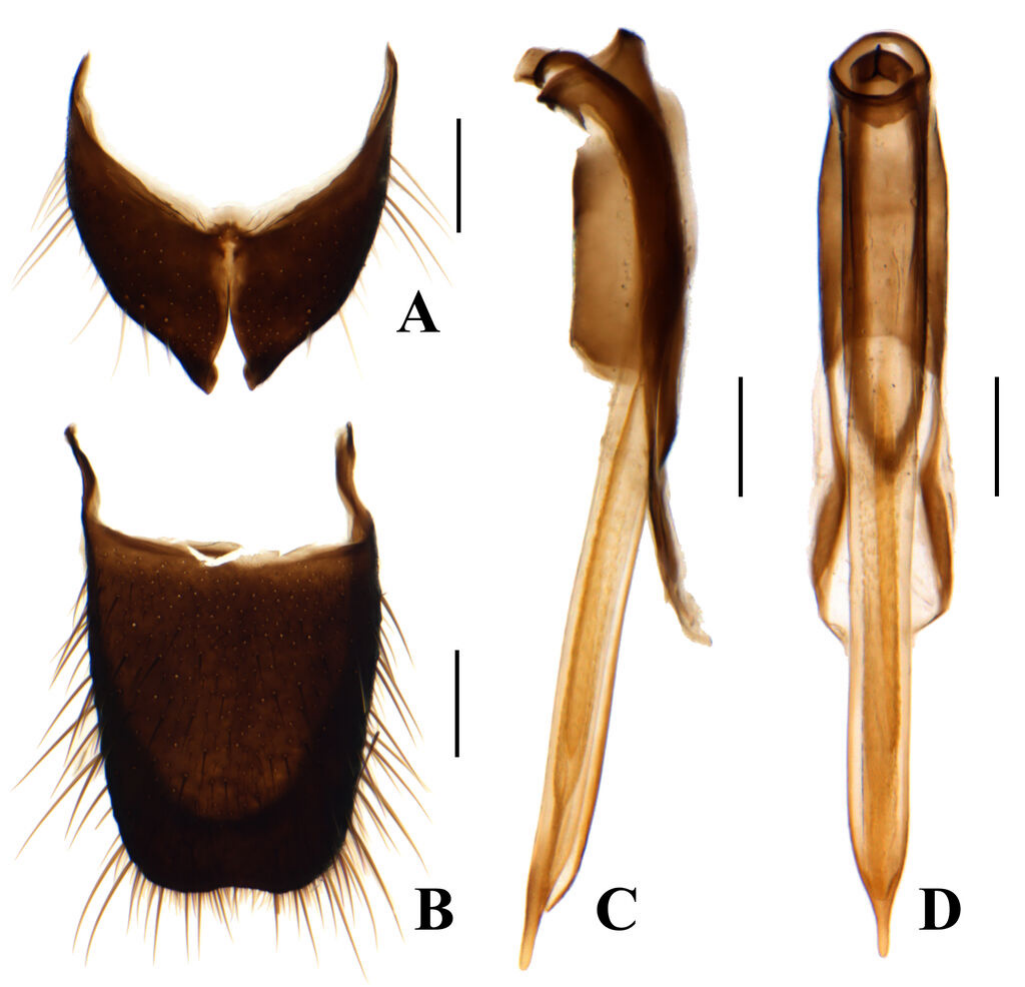
*Anthomalachiuspseudospinosus* (Medvedev, 1964), male: **A** ultimate abdominal ventrite; **B** pygidium; **C** male genitalia, lateral view; **D** male genitalia, ventral view. Scale bars: 0.2 mm.

Metathorax simple, lacking appendages. Pygidium rectangular, with apical margin emarginate (Fig. 4B); ultimate abdominal ventrite narrowly connected at middle with very deep apical incision (Fig. 4A). Tegmen elongate; aedeagus slender with lateral margins nearly parallel, apex with a distinct protuberance in ventral view (Fig. 4C and D).

**Female.** Length of body 4.2–5.6 mm, width at widest part of elytra 1.5–2.0 mm and at the base of elytra 1.1–1.3 mm.

Similar to male species, except for antennae thinner, interocular depression feeble and elytral apices simple without lamellate appendages (Fig. 1B).

**Figure 5.**
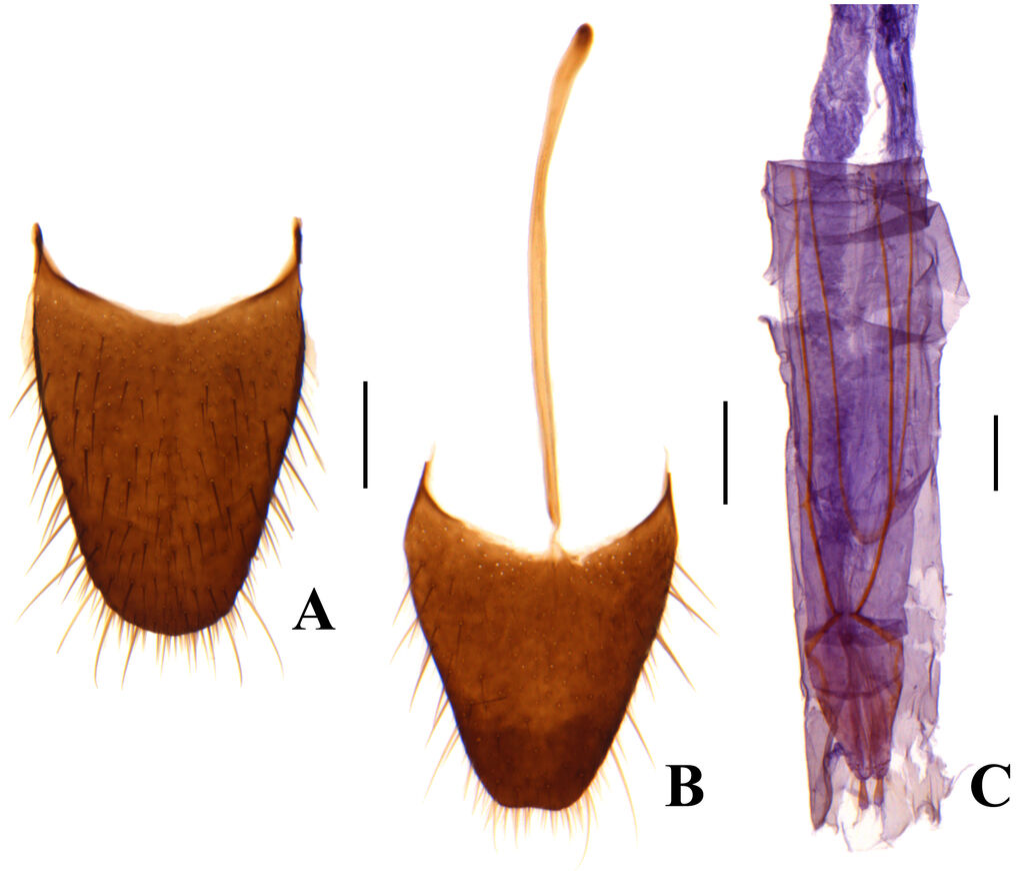
*Anthomalachiuspseudospinosus* (Medvedev, 1964), female: **A** pygidium; **B** ultimate abdominal ventrite; **C** ovipositor. Scale bars: 0.2 mm.

Pygidium large and elongate (Fig. 5A). Ultimate abdominal ventrite with long spiculum ventrale (Fig. 5B). Ovipositor elongate and membranous (Fig. 5C).

#### Diagnosis

This species closely resembles *A.senylia* (Tshernyshev, 2000), but can be distinguished from the latter by the thinner antennae, pygidium with apical margin emarginate in males and aedeagus with a distinct apical protuberance in ventral view. In *A.senylia*, antennae are more robust, pygidium slightly extending outwards in males and aedeagus is without protuberance (Tshernyshev 2000, Tshernyshev 2009).

#### Distribution

**Figure 6.**
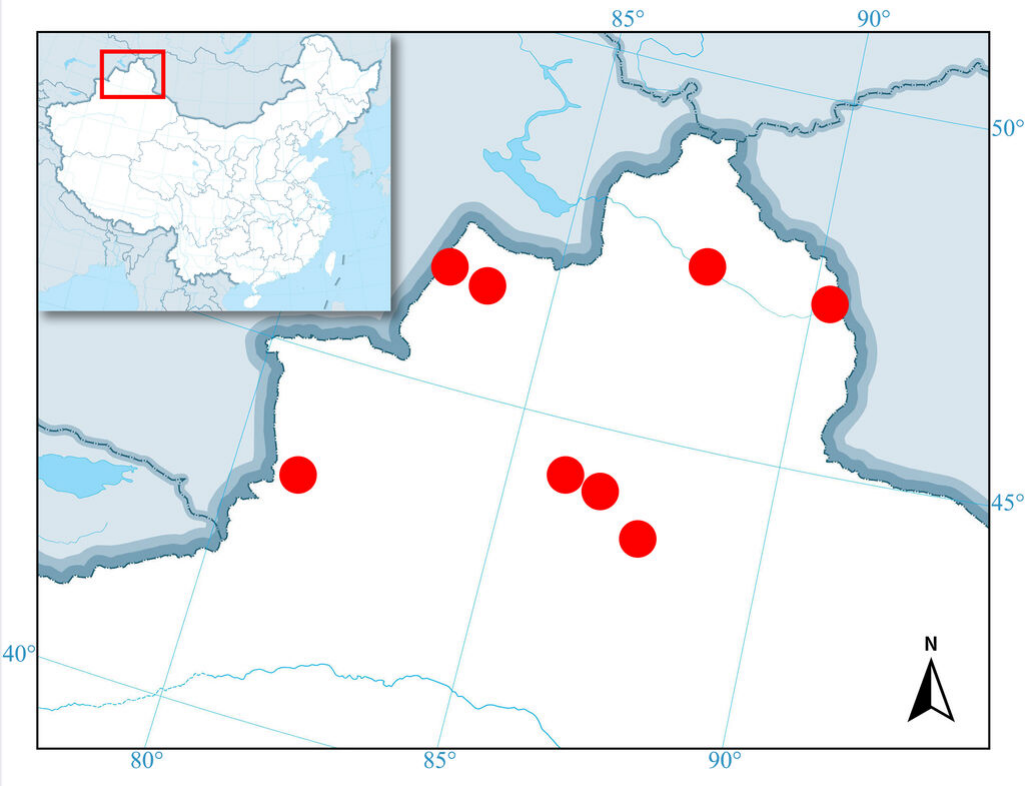
Distribution map of *Anthomalachiuspseudospinosus* (Medvedev, 1964) in Xinjiang Autonomous Region of China (red circle).

China (Xinjiang) (Fig. 6); Kazakhstan; Turkey.

## Supplementary Material

XML Treatment for Anthomalachius

XML Treatment for Anthomalachius pseudospinosus
